# Five-Year Efficacy and Safety of TiNO-Coated Stents Versus Drug-Eluting Stents in Acute Coronary Syndrome: A Meta-Analysis

**DOI:** 10.3390/jcm12216952

**Published:** 2023-11-06

**Authors:** Frederic C. Daoud, Bogdan Catargi, Pasi P. Karjalainen, Edouard Gerbaud

**Affiliations:** 1 Endocrinology-Metabolic Diseases, Hôpital Saint-André, Bordeaux University, 33000 Bordeaux, France; frederic.daoud-pineau@u-bordeaux.fr (F.C.D.); bogdan.catargi@chu-bordeaux.fr (B.C.); 2 Cardiac Unit, Heart and Lung Center, Helsinki University Hospital, Helsinki University, 00280 Helsinki, Finland; pasi.karjalainen@hus.fi; 3 Cardiology Intensive Care Unit and Interventional Cardiology, Hôpital Cardiologique du Haut-Lévêque, 33604 Pessac, France; 4 Bordeaux Cardio-Thoracic Research Centre, U1045, Bordeaux University, 33076 Bordeaux, France

**Keywords:** acute coronary syndrome, non-drug-eluting titanium-nitride-oxide-coated stents (TiNOS), drug-eluting stents, 5-year follow-up, safety, efficacy, systematic literature review, meta-analysis

## Abstract

(1) Background: Percutaneous coronary interventions (PCI) in patients with acute coronary syndrome (ACS) are performed with titanium-nitride-oxide-coated stents (TiNOSs) or drug-eluting stents (DESs). The initial completion of this prospective systematic literature review (SLR) of prospective randomized controlled trials (RCTs) showed that TiNOSs are non-inferior to DESs in major adverse cardiac event (MACE) rates and present a lower risk of recurrent myocardial infarction (MI) at 1-year follow-up. This iteration of the SLR protocol performs the critical assessment of 5-year follow-up outcomes with clinical validity and generalizability assessments. (2) Methods: The previously described SLR and meta-analysis protocol, per PRISMA, Cochrane methods, and GRADE, was applied to 5-year follow-up outcomes. (3) Results: Three RCTs were eligible, comprising 1620 patients with TiNOS vs. 1123 with DES. The pooled risk ratios (RRs) and 95% confidence intervals were MACE 0.82 [0.68, 0.99], MI 0.58 [0.44, 0.78], cardiac death (CD) 0.46 [0.28, 0.76], ischemia-driven target lesion revascularization (TLR) 1.03 [0.79, 1.33], probable or definite stent thrombosis (ST) 0.32 [0.21, 0.59], and all-cause mortality (TD) 0.84 [0.63, 1.12]. The evidence certainty was high in MACE, CD, MI, and ST, and moderate in TLR and TD. (4) Conclusions: TiNOSs in ACS at 5-year follow-up appear safer than DESs and equally efficacious. The pooled RRs stratified by clinical presentation and stent type will be required to test this meta-analysis’s clinical validity and generalize its results to patient populations with varying proportions of clinical presentations and DES options.

## 1. Introduction

Acute coronary syndrome (ACS) describes sudden myocardial ischemia. It comprises three clinical presentations: ST-segment-elevation myocardial infarction (STEMI), non-ST-segment-elevation myocardial infarction (NSTEMI), and unstable angina pectoris (UAP) [1]. This prospective systematic literature review (SLR) and meta-analysis of prospective randomized controlled trials (RCTs) compares the 1-year follow-up clinical outcomes after percutaneous coronary intervention (PCI) using non-drug-eluting titanium-nitride-oxide-coated stents (TiNOSs) to treat patients with ACS [2]. The pooled risk ratios (RRs) showed that TiNOSs were non-inferior to DESs in terms of device-oriented major adverse cardiac event (MACE) rates and presented a lower risk of recurrent myocardial infarction (MI). Five-year follow-up data were also searched, but the critical analysis and interpretation were postponed because one RCT with 54% of the required data was ongoing, which caused a major publication bias. The clinical validity and generalizability assessments were also delayed. The SLR was repeated and carried through with a focus on 5-year follow-up after all previously identified RCTs were completed and published.

## 2. Materials and Methods

This SLR was conducted according to a prospective protocol described in detail previously (PROSPERO CRD4201809062) and the two amendments below [2,3]. In brief, the research question was specified according to the PICO framework [4]. The SLR was conducted according to the principles described in the Cochrane Handbook and the “Preferred Reporting Items for Systematic Reviews and Meta-Analyses” (PRISMA) [5,6]. The primary outcome was cumulative device-oriented major adverse cardiac events (MACEs) up to 5-year follow-up. The secondary outcomes were cumulative cardiac death (CD), recurrent non-fatal myocardial infarction (MI), ischemia-driven target lesion revascularization (TLR), probable or definite stent thrombosis (ST), and all-cause mortality (“total death”, TD). The endpoint definitions were those of the Academic Research Consortium (ARC-2) [7].

Two reviewers, F.D. and B.C., separately searched, reviewed, classified, bias-risk-rated, and summarized the identified publications. E.G. adjudicated disagreements and P.K. obtained checked data in case of uncertainty.

Prospective RCTs comparing the above endpoints in patients with ACS at 5-year follow-up post-stenting with TiNOSs vs. DESs were searched in PubMed, Scopus, the Cochrane Library, and Web of Science (WoS) electronic databases on 5 July 2023, reusing first round’s search string [2]. The string was interpreted by each database’s search engine (Appendix A).

To avoid deriving conclusions about patients with chronic coronary syndrome (CCS) based on data from patients presenting with ACS, the RCT eligibility was narrowed down to trials that either included patients with ACS only or included a mix of patients with ACS and CCS, providing that the baseline and outcome data in ACS and CCS were reported separately. RCTs that did not report 5-year outcomes stratified by ACS and CCS were excluded.

All identified references were pooled and classified in EndNote (version X8. EndNote X9. Clarivate, Philadelphia, PA, USA). The data were extracted and copied into standard tables in Review Manager (RevMan Computer program. Version 5.3. Copenhagen, Denmark: The Nordic Cochrane Centre, The Cochrane Collaboration, 2014). Individual study bias was rated using RevMan’s seven-item instrument and operator blinding as a custom eight-item tool [8].

Mantel–Haenszel (M-H) fixed-effect pooled RRs and 95% confidence intervals (CIs) were computed for each endpoint with the event rate in TiNOS over the rate in DES on an intention-to-treat (ITT) basis. The results are displayed as Forest plots. If the authors reported event rates only, event numbers were back-calculated from the sample sizes. The publication bias of each outcome was assessed by searching the asymmetry of the funnel plot of the study RRs and Harbord’s regression test for the small-study effect in binary variables (significant if *p* < 0.05) [9,10,11,12]. No stratified analysis or meta-regression was performed. The heterogeneity of each outcome’s pooled RRs was tested with Cochran’s Q-test (significant if *p* < 0.10). Its magnitude was estimated with the I^2^ index using four heterogeneity thresholds: 0% to 40%, probably unimportant heterogeneity; 30% to 60%, moderate; 50% to 90%, substantial; and 75% to 100%, considerable [6]. Robustness to sensitivity analysis of each pooled RR was performed by iteratively recalculating it after removing one eligible RCT at a time. Pooled RR calculations, heterogeneity estimates, Forest plots, and funnel plots were performed in RevMan. Habord tests were computed in STATA (version 17, StataCorp LP, College Station, TX, USA) using the metan and metabias packages.

The certainty of the evidence was assessed according to the “Grading of Recommendations Assessment, Development, and Evaluation” (GRADE) in GRADEpro GDT software 2023 online (https://gradepro.org) (accessed on 31 August 2023) [13,14,15,16,17,18]. To improve the clinical intuitiveness of the pooled RR imprecision rating, GRADE’s 2011 guideline 6 was replaced with the minimally contextualized approach (guidance 34, 2022) [17,19]. Depending on the situation, an outcome’s pooled RR was imprecise when its CI crossed threshold lines of appreciable harm, benefit, or futility. The lower and upper RR thresholds were 0.9 and symmetrically 1.11 (i.e., 1/0.9). A relative risk reduction (RRR) of 10%, the “futility threshold” (lower threshold RR = 1 − RRR) in the Cochrane review of DES vs. BMS in ACS, defined the thresholds [20]. In MACE and TLR, where DES was assumed to be superior to TiNOS, the RR of events in TiNOS over DES was tested for non-inferiority, and the CI was rated seriously imprecise if it crossed the upper threshold. Safety outcome RRs (CD, MI, TD, and ST) underwent a two-sided superiority test with the usual null hypothesis of no difference between the event rates of the compared treatments. A significant RR’s CI was rated seriously imprecise if it crossed the nearest threshold. Non-significant RR CIs crossing any threshold were rated seriously imprecise.

Assessing the clinical validity of the pooled RR of one outcome variable requires checking that the observed rate of that outcome with each treatment in each clinical subgroup, contributing to the pooled RR, is consistent with the benchmark rates. The benchmarks are rates reported in studies as valid population estimates. Here, validation benchmarks were needed for each analyzed outcome variable with each stent type and each clinical presentation of ACS at 5-year follow-up.

Generalizing the pooled RR of one outcome variable requires checking that the target populations have the same background event risk for that variable as patient samples included in the pooled analysis. The comparison requires the main background factors strongly associated with risk. Multicollinearity between risk factors must be avoided.

## 3. Results

### 3.1. Study Identification and Selection

**Figure 1:**
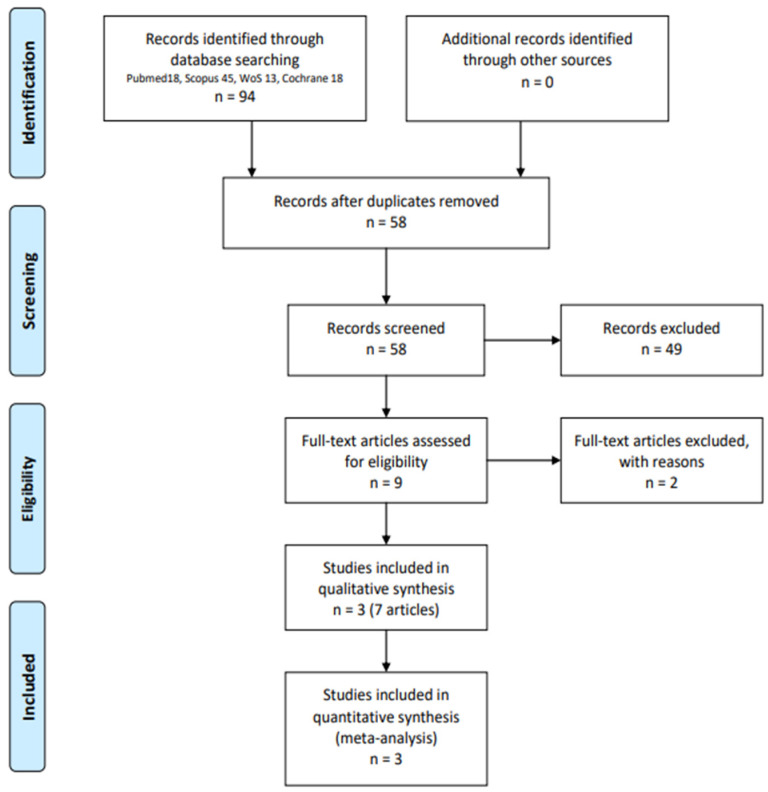
PRISMA flowchart.

Ninety-four records were identified, and nine publications with first-hand data regarding three RCTs were found to be eligible for pooling after selection (PRISMA flowchart in Figure 1) [5]. The 49 excluded records did not meet the PICO specifications or were review articles. One RCT comparing TiNOS to zotarolimus-eluting stents included 53% with CCS and 47% with ACS, but the study was excluded from the pooled analysis because it did not stratify the data [21,22].

### 3.2. Individual Study Characteristics

Table 1 summarizes the characteristics of the eligible RCTs and the reported raw data. The overall numbers and rates of patients lost to follow-up were 4.63% (67/1620) in the TiNOS arm and 4.36% (49/1123) in the DES arm. The pooled distribution of clinical presentations was NSTEMI 48.2%, STEMI 43.2%, and UAP 8.6%.

The previously reported number of patients by clinical presentation was inconsistent with the total sample size in the baseline characteristics of TIDES-ACS [23]. The authors provided the following corrigendum via P.K.: the number of patients with UAP was 87 in the TiNOS arm and 37 in the DES arm, not 126 and 61, respectively.

**Table 1:** Eligible studies for 5-year follow-up. Baseline characteristics.

Study	Age and Prior Events			Clinical Presentation (Included and Pooled)			Procedural Data and Medication			Lost to Follow-Up
Stent		TiNOS	DES		TiNOS N Incl, % pooled	DES N Incl, % pooled		TiNOS	DES	
TITAX-AMI DES = PES [24,25]	Patients n age prior MI prior PCI prior CABG	214 64 ± 11 15% 10% 7%	211 64 ± 11 9% 5% 6%	STEMI NSTEMI UAP	83, 39% 131, 61% 0, 0%	97, 46% 114, 54% 0, 0%	Stents/culprit lesion n TSL (mm) Post-dilation Procedural success DAPT 12 m	1.1 ± 0.3 18.5 ± 6.4 42% 99.5% 31%	1.1 ± 0.4 19.2 ± 7.2 35% 98.1% 65%	TiNOS: 3 DES: 7
BASE-ACS DES = EES [26,27]	Patients n age prior MI prior PCI prior CABG	417 63 ± 12 13.4% 9.6% 4.8%	410 63 ± 12 9.8% 10.5% 4.1%	STEMI NSTEMI UAP	162, 38.8% 206, 49.4% 49, 11.8%	159, 38.8% 187, 45.6% 64, 15.6%	Stents/culprit lesion n TSL (mm) Post-dilation Stent failure DAPT: Aspirin: N.R. Clopidogrel: N.R.	1.15 ± 0.38 20.8 ± 9.4 42.2% 0.0%	1.14 ± 0.36 20.6 ± 8.2 43.9% 1.0%	TiNOS: 29 DES: 28
TIDES-ACS DES = EES [23,28]	Patients n age prior MI prior PCI prior CABG	989 62.7 ± 10.7 6% 7.0% 0.6%	502 62.6 ± 10.5 9.0% 6.6% 1.2%	STEMI NSTEMI UAP	444, 44.9% 458, 46.3% 87, 8.8%	239, 47.6% 226, 45.8% 37, 7.4%	Stents/culprit lesion n TSL (mm) Post-dilation Stent failure DAPT 12 m	1.13 ± 0.38 20.5 ± 7.8 33.0% 0.3% 80.3%	1.14 ± 0.37 20.6 ± 7.2 38.0% 1.0% 86.0%	TiNOS: 35 DES: 14

EES: everolimus-eluting stent; PES: paclitaxel-eluting stent; N.R.: not reported; DAPT: dual antiplatelet treatment; TSL: total stent length.

### 3.3. Individual Study Risk of Bias

**Figure 2:**
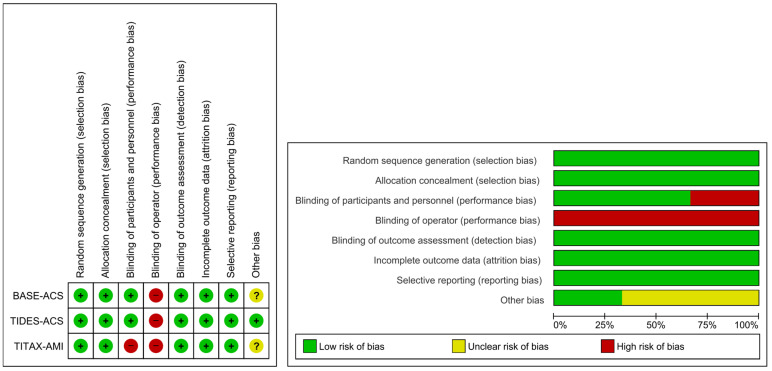
Individual study risk of bias—5-year follow-up.

The Cochrane instrument was used to rate the individual study 5-year follow-up risk of bias. The RCTs’ risk of bias was low, except for the consistent risk related to the operators’ knowledge of the stents they used (Figure 2).

### 3.4. Publication Bias Risk

**Figure 3:**
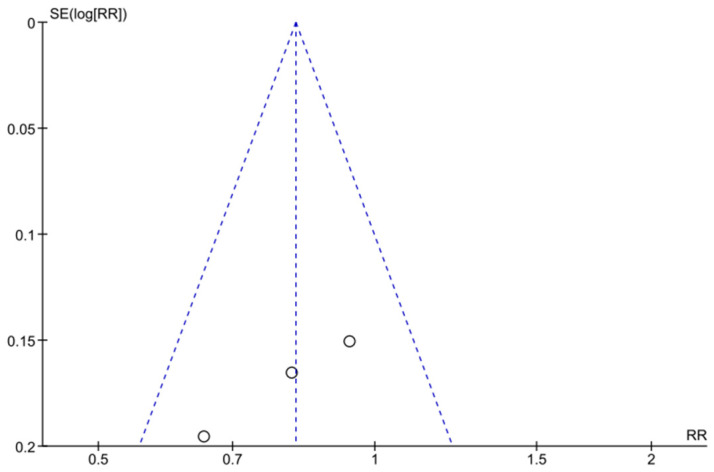
Funnel plot—risk of publication bias. Five-year cumulative MACE RR.

The 5-year MACE RR funnel plot (Figure 3) detected no risk of publication bias, and the Harbord test detected no small-study effect (*p* = 0.08).

Similar tests were applied to all other outcomes, and none was detected using funnel plots or Harbord tests. No small-study effect was detected with 5-year cumulative CD (*p =* 0.637), MI (*p =* 0.204), TLR (*p =* 0.277), ST (*p* = 0.085), or TD (*p* = 0.832).

### 3.5. Pooled Cumulative Outcomes

**Figure 4:**
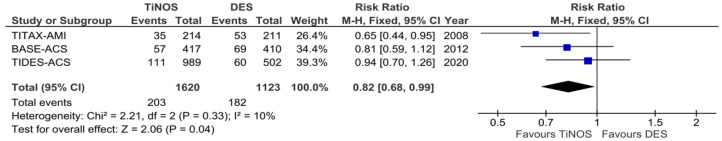
Forest plot—cumulative MACE. Five-year follow-up.

**Figure 5:**
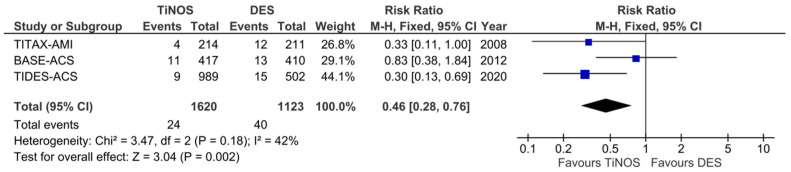
Forest plot—cumulative CD. Five-year follow-up.

**Figure 6:**
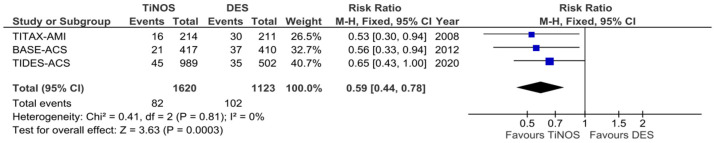
Forest plot—cumulative MI. Five-year follow-up.

**Figure 7:**
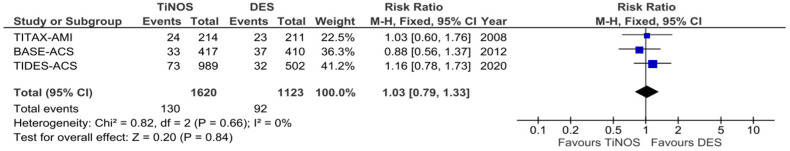
Forest plot—cumulative TLR. Five-year follow-up.

**Figure 8:**
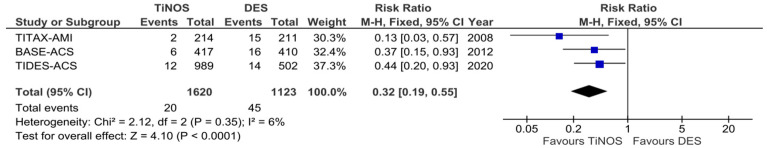
Forest plot—cumulative ST. Five-year follow-up.

**Figure 9:**
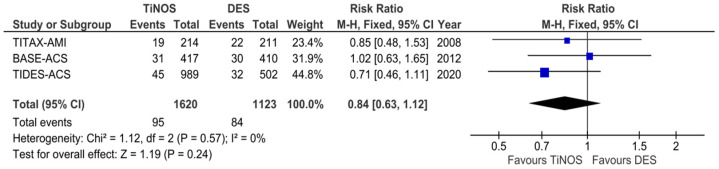
Forest plot—cumulative TD. Five-year follow-up.

Figure 4, Figure 5, Figure 6, Figure 7, Figure 8 and Figure 9 display the cumulative MACE-, CD-, MI-, TLR-, ST-, and TD-pooled RRs at 5-year follow-up. No significant heterogeneity was identified using Q-tests and I^2^ indices. The pooled RRs of the MI, CD, and ST rates show a significantly lower event rate with TiNOS than with DES. The pooled RRs of the TLR and MACE rates show TiNOS’ non-inferiority compared with DES, although the MACE rate with TiNOS was significantly lower than that with DES.

### 3.6. Sensitivity Analysis

The sensitivity analysis shows the impact on the pooled 5-year RRs of withdrawing one RCT at a time (Table 2). The pooled RRs of all endpoints, except those of MACE and CD, were robust to sensitivity analysis.

### 3.7. GRADE Certainty of Evidence

The GRADE rating (Table 3) showed high certainty levels with MACE, CD, MI, and probable or definite ST, and moderate levels with TLR and TD.

## 4. Discussion

### 4.1. Summary of the Results

The pooled analysis shows TiNOS’ non-inferiority at 5-year follow-up in MACE and superiority in MI, CD, and probable or definite ST, with a high level of certainty. It also shows the non-inferiority of TiNOS in TLR at a 5-year follow-up compared with a significantly higher rate of events than with DES at a 1-year follow-up, with a moderate certainty level due to the limited number of observations. TD’s RR was non-significant, but the certainty level was moderate for the same reasons as TLR.

The increase in cumulative events in the two compared arms from one- to five-year follow-ups and the updated precision grading method contributed to the increased certainty of outcomes, although the RR estimates of some outcomes still had limited precision [17]. An extended follow-up of the patients beyond five years, if possible, would help to identify further changes in all outcome variables, including TLR.

Overall, the 5-year results show the long-term superior safety of TiNOS over DES in ACS with a high level of certainty. The evidence also shows a progressive efficacy shift with the TLR rate that was significantly higher in TiNOS vs. DES at 1-year follow-up and reached non-inferiority at 5-year follow-up, although the number of observations remained insufficient for the level of evidence to become high. Extended follow-ups beyond five years should be monitored to check whether the shift continues over time.

The low rate of patients lost to follow-up in each RCT contributed to the continued low risk of bias at 5-year follow-up.

### 4.2. Clinical Validity

The clinical validity of the pooled RRs requires checking that the 5-year MACE, CD, MI, TLR, probable or definite ST, and TD rates in ACS with TiNOS, EES, and PES are consistent with benchmark rates regarded as valid, reported in samples of patients presenting with STEMI, NSTEMI, and UAP treated with the same stent types with 5-year follow-up as well. A PubMed and Cochrane database search for RCTs or meta-analyses of RCTs with the specifications described above returned no evidence for TiNOS, but two meta-analyses comparing DES vs. BMS.

One individual patient data meta-analysis (IPDM) pooled data from 14 RCTs comparing DES and BMS in ACS [29]. The results were stratified outcome ratios up to 5-year follow-up in 34.5% of patients with CCS and 65.5% of patients with ACS. In the ACS subgroup, 7739 patients were assigned to DES vs. 6889 to BMS. The source publications of the included studies were reviewed, and ACS clinical presentations, DES types, outcome definitions, sample sizes, and follow-up durations were checked [30,31,32,33,34,35,36,37,38,39,40,41,42,43,44,45]. None of the RCTs provided validation benchmarks for the DES arm.

One Cochrane review pooled 25 RCTs comparing the outcomes of DES vs. BMS in ACS [20]. The source publications were checked using the same methods as the IPDM [46,47,48,49,50,51,52,53,54,55,56,57,58,59,60,61,62,63,64,65,66,67,68,69,70,71]. Overall, 6916 patients were treated with DES vs. 5640 with BMS. A single RCT included patients with NSTEMI treated with EES, but the follow-up duration was only two years [61]. Another RCT included patients with STEMI treated with EES and reported outcomes at 5-year follow-up [46]. Another RCT included patients with STEMI treated with stainless-steel paclitaxel-eluting stents (PES) or BMS and reported outcomes at 5-year follow-up [67]. The latter two RCTs were the Cochrane review’s potential contribution to the validation benchmarks of the DES arm.

This meta-analysis comparing outcomes at five years with TiNOS vs. DES in ACS is the first of its kind. Therefore, clinical validation will require pooling additional future evidence to compare each stent and each clinical presentation with the corresponding benchmarks.

### 4.3. Generalization of the Results

Generalizing the results of this meta-analysis to a population requires either the overall similarity of background risk factors in the pooled study sample and the target population, in which case the overall pooled RR can be generalized, or similar subgroups with different proportions. Then, each stratified pooled RR can be generalized to the corresponding population subgroup. ACS covers the diversity of clinical presentations in emergency coronary care. Several epidemiological surveys have consistently shown that the incidence rate of STEMI decreases with age. In contrast, the incidence rate of NSTEMI increases with equal rates at around 65 years of age, women present more frequently with NSTEMI or UAP than STEMI, and patients with STEMI are more often smokers than patients with NSTEMI [72,73,74,75,76]. Therefore, the PCI outcomes at 5-year follow-up in any study should logically be associated with those baseline characteristics and other factors, such as emergency care availability. A panel of epidemiological surveys on ACS hospital admissions and reperfusion rates shows significant differences in STEMI, NSTEMI, and UAP proportions across the sampled populations [72,73,76,77,78,79,80]. These differences show the difficulty of directly applying the outcomes of the three pooled RCTs of this meta-analysis to populations with a different case mix. Pooled RRs stratified by clinical presentation and stent type will be required to generalize their results to populations with varying proportions of clinical presentations.

### 4.4. Plaque Destabilization Risk

In addition to the population’s baseline characteristics and the different clinical presentations, the abrupt occurrence of ACS is a strong signal of discontinuity in the natural history of atherosclerosis. The causes of this discontinuity are complex and unknown to a large extent. Understanding them is necessary to identify new treatment targets. Proprotein convertase subtilisin/kexin type 9 (PCSK9) has been identified as a key orchestrator of the atherosclerotic process [81]. Regarding the mechanism, PCSK9 plays multiple immune roles in platelet aggregation, adhesion to endothelial cells, and endothelial dysfunctions. PCSK9 inhibitors significantly decrease low-density lipoprotein cholesterol (LDL-C) levels by reducing LDL receptor degradation and have demonstrated their safety. Recent guidelines recommend PCSK9 inhibitors during ACS hospitalization in patients who fail to achieve target lipid levels despite receiving statin and ezetimibe treatments before admission [82]. Studies now explore the optimal timing of PCSK9 inhibitor use in patients who present with ACS. The PACMAN-AMI study suggests that early PCSK9 inhibitors can slow down plaque progression and reduce the short-term risk of cardiovascular events [83].

Additionally, systemic or regional inflammation can hasten plaque changes and promote atherosclerosis progression with complications, including ACS [84]. Cytokines and chemokines, including CXCL12, IL-8, and IL-6, are associated with ACS occurrence [85]. The higher levels of CXCL12 in atherosclerotic lesions than in healthy vessel wall cells suggest that CXCL12 is associated with cardiovascular diseases. The clinical significance of platelets’ increased CXCL12 expression in patients with angina pectoris remains to be clarified [86]. IL-8 is produced by various cell types involved in atherosclerosis and expressed in areas of atherosclerotic lesions rich in macrophages. IL-8 can contribute to atherosclerotic complications through thrombosis and plaque destabilization [87]. Finally, the vulnerability of the plaque is enhanced when macrophage efferocytosis results are impaired, with a consequent switch in cytokine secretion from a pro-resolution to pro-inflammatory fashion, including TNF-α, IL-1β, and IL-6. These mediators can stimulate vascular smooth muscle cells, through nuclear factor-kB pathways, to release matrix metalloproteinases and other inflammatory genes, which contributes to reducing the stability of the plaque, thus aggravating the necrotic core and thinning the fibrous cap [88]. Thus, it is important to explore ways to modulate the immune response to reduce inflammation in the arterial walls. In the CANTOS trial, the patients were randomized to receive IL-1β inhibitors (i.e., Canakinumab) versus placebo, and those treated with the former at a subcutaneous dose of 150 mg once every 3 months had a significantly lower rate of recurrent cardiovascular events than those receiving the placebo, independent of lipid-level lowering [89].

## 5. Conclusions

The pooled outcomes at five-year follow-up show that titanium-nitride-oxide-coated stents are safer than drug-eluting stents in patients with acute coronary syndrome. The risk of cardiac death, myocardial infarction, and stent thrombosis is significantly lower with titanium-nitride-oxide-coated stents than those with paclitaxel or everolimus-eluting metallic stents, with a high certainty level. The two stents display similar efficacy with non-significantly different target lesion revascularization rates and a moderate certainty level. All-cause mortality rates are not significantly different, with an intermediate certainty level. Overall, the rates of device-oriented major adverse cardiac events are not significantly different, with a high certainty level. These pooled results are the first of their kind. Pooled relative risks stratified by clinical presentation and stent type will be required to test this meta-analysis’s clinical validity against external benchmarks and to generalize its results to patient populations with varying proportions of acute coronary clinical presentations and drug-eluting stent options.

The references identified as ineligible for the meta-analysis are listed in Appendix B.

## Figures and Tables

**Table 2:** Sensitivity analysis of all endpoints for TiNOS vs. DES at 5-year follow-up.

M-H Fixed-Effects RR and 95% CI after the Removal of:						
Outcome	None	TITAX-AMI	TIDE	BASE-ACS	TIDES-ACS	Robustness
MACE	0.82 [0.68, 0.99]	0.88 [0.71, 1.09]	N.A.	0.82 [0.65, 1.04]	0.74 [0.58, 0.95]	No
CD	0.46 [0.28, 0.76]	0.51 [0.30, 0.89]	N.A.	0.31 [0.16, 0.61]	0.59 [0.31, 1.11]	No
MI	0.59 [0.44, 0.78]	0.61 [0.44, 0.85]	N.A.	0.60 [0.43, 0.85]	0.54 [0.37, 0.80]	Yes
TLR	1.03 [0.79, 1.33]	1.03 [0.76, 1.38]	N.A.	1.11 [0.81, 1.54]	0.94 [0.66, 1.32]	Yes
probable or definite ST	0.32 [0.19, 0.55]	0.40 [0.22, 0.73]	N.A.	0.30 [0.15, 0.58]	0.25 [0.12, 0.55]	Yes
TD	0.84 [0.63, 1.12]	0.84 [0.61, 1.16]	N.A.	0.76 [0.54, 1.08]	1.03 [0.74, 1.45]	Yes

N.A.: Not applicable.

**Table 3:** GRADE summary of findings—TiNOS vs. DES in ACS at 5-year follow-up.

Outcome	Risk of Bias	Inconsistency	Indirectness	Imprecision	Publication Bias	Certainty of Evidence
MACE	not serious	not serious	not serious	not serious ^a^	none	⨁⨁⨁⨁ HIGH
CD	not serious	not serious ^c^	not serious	not serious ^b^	none	⨁⨁⨁⨁ HIGH
MI	not serious	not serious	not serious	not serious ^b^	none	⨁⨁⨁⨁ HIGH
TLR	not serious	not serious	not serious	serious ^d^	none	⨁⨁⨁◯ MODERATE
probable or definite ST	not serious	not serious	not serious	not serious ^b^	none	⨁⨁⨁⨁ HIGH
TD	not serious	not serious	not serious	serious ^e^	none	⨁⨁⨁◯ MODERATE

CI: confidence interval; RR: risk ratio. ^a^ The CI does not cross the RR = 1.11 threshold. ^b^ The CI does not cross the RR = 0.9 and RR = 1.11 thresholds. ^c^ Although I^2^ = 42% and Q-test *p* = 0.18, the three-point estimates of RR are <1 with overlapping CIs. ^d^ The CI crosses the RR = 1.11 threshold. ^e^ The CI crosses the RR = 0.9 and RR = 1.11 thresholds.

## Data Availability

Not applicable. This work uses publicly available peer-reviewed articles.

## Appendix A. Detailed Search Strings in Each Database

PubMed search string: *((bioactive OR (Titanium AND nitride AND oxide) OR TiNO OR TNO OR BAS) AND stent) AND (DES OR (drug AND eluting AND stent)) AND (RCT OR ((randomised OR randomised) AND controlled AND trial))*

*(“bioactivate”[All Fields] OR “bioactivated”[All Fields] OR “bioactivates”[All Fields] OR “bioactivating”[All Fields] OR “bioactivation”[All Fields] OR “bioactivations”[All Fields] OR “bioactive”[All Fields] OR “bioactives”[All Fields] OR “bioactivities”[All Fields] OR “bioactivity”[All Fields] OR ((“titanium”[MeSH Terms] OR “titanium”[All Fields] OR “titaniums”[All Fields]) AND (“nitridated”[All Fields] OR “nitridation”[All Fields] OR “nitride”[All Fields] OR “nitrided”[All Fields] OR “nitrides”[All Fields] OR “nitriding”[All Fields] OR “nitridized”[All Fields]) AND (“oxidability”[All Fields] OR “oxidable”[All Fields] OR “oxidant s”[All Fields] OR “oxidants”[Pharmacological Action] OR “oxidants”[MeSH Terms] OR “oxidants”[All Fields] OR “oxidant”[All Fields] OR “oxidate”[All Fields] OR “oxidated”[All Fields] OR “oxidates”[All Fields] OR “oxidating”[All Fields] OR “oxidation”[All Fields] OR “oxidations”[All Fields] OR “oxidative”[All Fields] OR “oxidatively”[All Fields] OR “oxidatives”[All Fields] OR “oxide s”[All Fields] OR “oxides”[MeSH Terms] OR “oxides”[All Fields] OR “oxide”[All Fields] OR “oxidic”[All Fields] OR “oxiding”[All Fields] OR “oxidisability”[All Fields] OR “oxidisable”[All Fields] OR “oxidisation”[All Fields] OR “oxidise”[All Fields] OR “oxidised”[All Fields] OR “oxidiser”[All Fields] OR “oxidisers”[All Fields] OR “oxidises”[All Fields] OR “oxidising”[All Fields] OR “oxidization”[All Fields] OR “oxidize”[All Fields] OR “oxidized”[All Fields] OR “oxidizer”[All Fields] OR “oxidizers”[All Fields] OR “oxidizes”[All Fields] OR “oxidizing”[All Fields])) OR “TiNO”[All Fields] OR “TNO”[All Fields] OR “BAS”[All Fields]) AND (“stent s”[All Fields] OR “stentings”[All Fields] OR “stents”[MeSH Terms] OR “stents”[All Fields] OR “stent”[All Fields] OR “stented”[All Fields] OR “stenting”[All Fields]) AND (“DES”[All Fields] OR (“drug”[All Fields] AND (“elutable”[All Fields] OR “elutant”[All Fields] OR “elute”[All Fields] OR “eluted”[All Fields] OR “elutent”[All Fields] OR “eluter”[All Fields] OR “eluters”[All Fields] OR “elutes”[All Fields] OR “eluting”[All Fields] OR “elution”[All Fields] OR “elutions”[All Fields]) AND (“stent s”[All Fields] OR “stentings”[All Fields] OR “stents”[MeSH Terms] OR “stents”[All Fields] OR “stent”[All Fields] OR “stented”[All Fields] OR “stenting”[All Fields]))) AND (“RCT”[All Fields] OR ((“random allocation”[MeSH Terms] OR (“random”[All Fields] AND “allocation”[All Fields]) OR “random allocation”[All Fields] OR “random”[All Fields] OR “randomization”[All Fields] OR “randomized”[All Fields] OR “randomisation”[All Fields] OR “randomisations”[All Fields] OR “randomise”[All Fields] OR “randomised”[All Fields] OR “randomising”[All Fields] OR “randomizations”[All Fields] OR “randomize”[All Fields] OR “randomizes”[All Fields] OR “randomizing”[All Fields] OR “randomness”[All Fields] OR “randoms”[All Fields] OR (“random allocation”[MeSH Terms] OR (“random”[All Fields] AND “allocation”[All Fields]) OR “random allocation”[All Fields] OR “random”[All Fields] OR “randomization”[All Fields] OR “randomized”[All Fields] OR “randomisation”[All Fields] OR “randomisations”[All Fields] OR “randomise”[All Fields] OR “randomised”[All Fields] OR “randomising”[All Fields] OR “randomizations”[All Fields] OR “randomize”[All Fields] OR “randomizes”[All Fields] OR “randomizing”[All Fields] OR “randomness”[All Fields] OR “randoms”[All Fields])) AND “controlled”[All Fields] AND (“clinical trials as topic”[MeSH Terms] OR (“clinical”[All Fields] AND “trials”[All Fields] AND “topic”[All Fields]) OR “clinical trials as topic”[All Fields] OR “trial”[All Fields] OR “trial s”[All Fields] OR “trialed”[All Fields] OR “trialing”[All Fields] OR “trials”[All Fields])))*

*Translations*

*bioactive: “bioactivate”[All Fields] OR “bioactivated”[All Fields] OR “bioactivates”[All Fields] OR “bioactivating”[All Fields] OR “bioactivation”[All Fields] OR “bioactivations”[All Fields] OR “bioactive”[All Fields] OR “bioactives”[All Fields] OR “bioactivities”[All Fields] OR “bioactivity”[All Fields]*

*Titanium: “titanium”[MeSH Terms] OR “titanium”[All Fields] OR “titanium’s”[All Fields] OR “titaniums”[All Fields]*

*nitride: “nitridated”[All Fields] OR “nitridation”[All Fields] OR “nitride”[All Fields] OR “nitrided”[All Fields] OR “nitrides”[All Fields] OR “nitriding”[All Fields] OR “nitridized”[All Fields]*

*oxide: “oxidability”[All Fields] OR “oxidable”[All Fields] OR “oxidant’s”[All Fields] OR “oxidants”[Pharmacological Action] OR “oxidants”[MeSH Terms] OR “oxidants”[All Fields] OR “oxidant”[All Fields] OR “oxidate”[All Fields] OR “oxidated”[All Fields] OR “oxidates”[All Fields] OR “oxidating”[All Fields] OR “oxidation”[All Fields] OR “oxidations”[All Fields] OR “oxidative”[All Fields] OR “oxidatively”[All Fields] OR “oxidatives”[All Fields] OR “oxide’s”[All Fields] OR “oxides”[MeSH Terms] OR “oxides”[All Fields] OR “oxide”[All Fields] OR “oxidic”[All Fields] OR “oxiding”[All Fields] OR “oxidisability”[All Fields] OR “oxidisable”[All Fields] OR “oxidisation”[All Fields] OR “oxidise”[All Fields] OR “oxidised”[All Fields] OR “oxidiser”[All Fields] OR “oxidisers”[All Fields] OR “oxidises”[All Fields] OR “oxidising”[All Fields] OR “oxidization”[All Fields] OR “oxidize”[All Fields] OR “oxidized”[All Fields] OR “oxidizer”[All Fields] OR “oxidizers”[All Fields] OR “oxidizes”[All Fields] OR “oxidizing”[All Fields]*

*stent: “stent’s”[All Fields] OR “stentings”[All Fields] OR “stents”[MeSH Terms] OR “stents”[All Fields] OR “stent”[All Fields] OR “stented”[All Fields] OR “stenting”[All Fields]*

*eluting: “elutable”[All Fields] OR “elutant”[All Fields] OR “elute”[All Fields] OR “eluted”[All Fields] OR “elutent”[All Fields] OR “eluter”[All Fields] OR “eluters”[All Fields] OR “elutes”[All Fields] OR “eluting”[All Fields] OR “elution”[All Fields] OR “elutions”[All Fields]*

*stent: “stent’s”[All Fields] OR “stentings”[All Fields] OR “stents”[MeSH Terms] OR “stents”[All Fields] OR “stent”[All Fields] OR “stented”[All Fields] OR “stenting”[All Fields]*

*randomized: “random allocation”[MeSH Terms] OR (“random”[All Fields] AND “allocation”[All Fields]) OR “random allocation”[All Fields] OR “random”[All Fields] OR “randomization”[All Fields] OR “randomized”[All Fields] OR “randomisation”[All Fields] OR “randomisations”[All Fields] OR “randomise”[All Fields] OR “randomised”[All Fields] OR “randomising”[All Fields] OR “randomizations”[All Fields] OR “randomize”[All Fields] OR “randomizes”[All Fields] OR “randomizing”[All Fields] OR “randomness”[All Fields] OR “randoms”[All Fields]*

*randomised: “random allocation”[MeSH Terms] OR (“random”[All Fields] AND “allocation”[All Fields]) OR “random allocation”[All Fields] OR “random”[All Fields] OR “randomization”[All Fields] OR “randomized”[All Fields] OR “randomisation”[All Fields] OR “randomisations”[All Fields] OR “randomise”[All Fields] OR “randomised”[All Fields] OR “randomising”[All Fields] OR “randomizations”[All Fields] OR “randomize”[All Fields] OR “randomizes”[All Fields] OR “randomizing”[All Fields] OR “randomness”[All Fields] OR “randoms”[All Fields]*

*trial: “clinical trials as topic”[MeSH Terms] OR (“clinical”[All Fields] AND “trials”[All Fields] AND “topic”[All Fields]) OR “clinical trials as topic”[All Fields] OR “trial”[All Fields] OR “trial’s”[All Fields] OR “trialed”[All Fields] OR “trialing”[All Fields] OR “trials”[All Fields]*

Cochrane database search string: *((bioactive OR (Titanium AND nitride AND oxide) OR TiNO OR TNO OR BAS) AND stent) AND (DES OR (drug AND eluting AND stent)) AND (RCT OR ((randomised OR randomised) AND controlled AND trial))*.

Web of Science search string: *(((bioactive OR (Titanium AND nitride AND oxide) OR TiNO OR TNO OR BAS) AND stent) AND (DES OR (drug AND eluting AND stent)) AND (RCT OR ((randomised OR randomised) AND controlled AND trial)))*

Embase search string: *(((bioactive OR (Titanium AND nitride AND oxide) OR TiNO OR TNO OR BAS) AND stent) AND (DES OR (drug AND eluting AND stent)) AND (RCT OR ((randomised OR randomised) AND controlled AND trial)))/br.*

## Appendix B. Identified References Ineligible for the Meta-Analysis

- Windecker, S.; Simon, R.; Lins, M.; Klauss, V.; Eberli, F.R.; Roffi, M.; Pedrazzini, G.; Moccetti, T.; Wenaweser, P.; Togni, M.; et al. Randomized comparison of a titanium-nitride-oxide-coated stent with a stainless steel stent for coronary revascularization—The TiNOX trial. *Circulation* **2005**, *111*, 2617–2622. https://doi.org/10.1161/CIRCULATIONAHA.104.486647.
- Grube, E.; Buellesfeld, L. BioMatrix Biolimus A9-eluting coronary stent: A next-generation drug-eluting stent for coronary artery disease. *Expert Rev. Med. Devices* **2006**, *3*, 731–741. https://doi.org/10.1586/17434440.3.6.731.
- Wu, P.; Grainger, D.W. Drug/device combinations for local drug therapies and infection prophylaxis. *Biomaterials* **2006**, *27*, 2467.
- Coolong, A.; Kuntz, R.E. Understanding the drug-eluting stent trials. *Am. J. Cardiol.* **2007**, *100*, S17–S24. https://doi.org/10.1016/j.amjcard.2007.06.004.
- Konorza, T.F.M. Prospective, multi-center randomized trial to compare the implantation of a titanium-nitride-oxide coated stent with a paclitaxel stent in patients with acute myocardial infarction. *Herz* **2007**, *32*, 513. https://doi.org/10.1007/s00059-007-3036-6.
- Karjalainen, P.P.; Ylitalo, A.; Niemelä, M.; Kervinen, K.; Mäkikallio, T.; Pietilä, M.; Sia, J.; Tuomainen, P.; Nyman, K.; Airaksinen, K.E.J. Two-year follow-up after percutaneous coronary intervention with titanium-nitride-oxide-coated stents versus paclitaxel-eluting stents in acute myocardial infarction. *Ann. Med.* **2009**, *41*, 599–607. https://doi.org/10.1080/07853890903111018.
- Sant’Anna, F.M.; Batista, L.A.; Brito, M.B.; Menezes, S.; Ventura, F.M.; Buczynski, L.; Barrozo, C.A.M. Randomized comparison of percutaneous coronary intervention with titanium-nitride-oxide-coated stents versus stainless steel stents in patients with coronary artery disease: RIO trial. *Rev. Bras. Cardiol. Invasia* **2009**, *17*, 69–75.
- Dibra, A.; Tiroch, K.; Schulz, S.; Kelbæk, H.; Spaulding, C.; Laarman, G.J.; Valgimigli, M.; Di Lorenzo, E.; Kaiser, C.; Tierala, I.; et al. Drug-eluting stents in acute myocardial infarction: Updated meta-analysis of randomized trials. *Clin. Res. Cardiol.* **2010**, *99*, 345–357. https://doi.org/10.1007/s00392-010-0133-y.
- Konorza, T.F.M. Randomized comparison of titanium-nitride-oxide coated stents with zotarolimus-eluting stents for coronary revascularisation. *Herz* **2010**, *35*, 364–367. https://doi.org/10.1007/s00059-010-3359-6.
- Moschovitis, A.; Simon, R.; Seidenstucker, A.; Klauss, V.; Baylacher, M.; Luscher, T.F.; Moccetti, T.; Windecker, S.; Meier, B.; Hess, O.M. Randomised comparison of titanium-nitride-oxide coated stents with bare metal stents: Five year follow-up of the TiNOX trial. *EuroIntervention* **2010**, *6*, 63–68.
- Karjalainen, P.; Nammas, W. Bioactive stents for percutaneous coronary intervention: A new forerunner on the track. *Intervent. Cardiol.* **2011**, *3*, 527–529. https://doi.org/10.2217/ica.11.61.
- Boden, H.; van der Hoeven, B.L.; Liem, S.S.; Atary, J.Z.; Cannegieter, S.C.; Atsma, D.E.; Bootsma, M.; Jukema, J.W.; Zeppenfeld, K.; Oemrawsingh, P.V.; et al. Five-year clinical follow-up from the MISSION! Intervention Study: Sirolimus-eluting stent versus bare metal stent implantation in patients with ST-segment elevation myocardial infarction, a randomised controlled trial. *EuroIntervention* **2012**, *7*, 1021–1029. https://doi.org/10.4244/EIJV7I9A164.
- Lehtinen, T.; Airaksinen, K.E.; Ylitalo, A.; Karjalainen, P.P. Stent strut coverage of titanium-nitride-oxide coated stent compared to paclitaxel-eluting stent in acute myocardial infarction: TITAX-OCT study. *Int. J. Cardiovasc. Imaging* **2012**, *28*, 1859–1866. https://doi.org/10.1007/s10554-012-0032-6.
- Tuomainen, P.O.; Ylitalo, A.; Niemelä, M.; Kervinen, K.; Pietilä, M.; Sia, J.; Nyman, K.; Nammas, W.; Airaksinen, K.E.J.; Karjalainen, P.P. Gender-based analysis of the 3-year outcome of bioactive stents versus paclitaxel-eluting stents in patients with acute myocardial infarction: An insight from the TITAX-AMI trial. *J. Invasive Cardiol.* **2012**, *24*, 104–108.
- De Luca, G.; Dirksen, M.T.; Spaulding, C.; Kelbk, H.; Schalij, M.; Thuesen, L.; Van der Hoeven, B.; Vink, M.A.; Kaiser, C.; Musto, C.; et al. Impact of Diabetes on Long-Term Outcome after Primary Angioplasty. *Diabetes Care* **2013**, *36*, 1020–1025.
- Karjalainen, P. Neointimal coverage and vasodilator response to titanium-nitride-oxide- coated bioactive stents and everolimus-eluting stents in patients with acute coronary syndrome: Insights from the BASE-ACS trial. *Int. J. Card. Imaging* **2013**, *29*, 1693–1703. https://doi.org/10.1007/s10554-013-0285-8.
- Lammer, J.; Zeller, T.; Hausegger, K.A.; Schaefer, P.J.; Gschwendtner, M.; Mueller-Huelsbeck, S.; Rand, T.; Funovics, M.; Wolf, F.; Rastan, A.; et al. Heparin-bonded covered stents versus bare-metal stents for complex femoropopliteal artery lesions: The randomized VIASTAR trial (viabahn endoprosthesis with propaten bioactive surface [VIA] versus bare nitinol stent in the treatment of long lesions in superficial femoral artery occlusive disease). *J. Am. Coll. Cardiol.* **2013**, *62*, 1320–1327. https://doi.org/10.1016/j.jacc.2013.05.079.
- Romppanen, H.; Nammas, W.; Kervinen, K.; Mikkelsson, J.; Pietilä, M.; Lalmand, J.; Rivero-Crespo, F.; Pentikäinen, M.; Tedjokusumo, P.; Karjalainen, P.P. Outcome of ST-elevation myocardial infarction versus non-ST-elevation acute coronary syndrome treated with titanium-nitride-oxide-coated versus everolimus-eluting stents: Insights from the BASE-ACS trial. *Minerva Cardioangiol.* **2013**, *61*, 201–209.
- Velders, M.A.; Boden, H.; van der Hoeven, B.L.; Liem, S.S.; Atary, J.Z.; van der Wall, E.E.; Jukema, J.W.; Schalij, M.J. Long-term outcome of second-generation everolimus-eluting stents and Endeavor zotarolimus-eluting stents in a prospective registry of ST-elevation myocardial infarction patients. *EuroIntervention* **2013**, *8*, 1206.
- Huang, Y.; Ng, H.C.A.; Ng, X.W.; Subbu, V. Drug-eluting biostable and erodible stents. *J. Control. Release* **2014**, *193*, 188–201. https://doi.org/10.1016/j.jconrel.2014.05.011.
- López-Mínguez, J.R.; Nogales-Asensio, J.M.; Doncel-Vecino, L.J.; Merchán-Herrera, A.; Pomar-Domingo, F.; Martínez-Romero, P.; Fernández-Díaz, J.A.; Valdesuso-Aguilar, R.; Moreu-Burgos, J.; Díaz-Fernández, J. A randomized study to compare bioactive titanium stents and everolimus-eluting stents in diabetic patients (TITANIC XV): 1-year results. *Rev. Esp. Cardiol. Engl. Ed.* **2014**, *67*, 522–530. https://doi.org/10.1016/j.rec.2013.10.021.
- Ribamar Costa, J.; Almeida, B.O.; Costa, R.; Chamié, D.; Abizaid, A.; Perin, M.; Staico, R.; Feres, F.; Siqueira, D.; Veloso, M.; et al. Comparison of drug-eluting stents with durable or bioabsorbable polymer: Intracoronary ultrasound results of the BIOACTIVE trial. *Rev. Bras. Cardiol. Invasia* **2014**, *22*, 245–251.
- Tuomainen, P.O.; Sia, J.; Nammas, W.; Niemelä, M.; Airaksinen, J.K.; Biancari, F.; Karjalainen, P.P. Pooled analysis of two randomized trials comparing titanium-nitride-oxide-coated stent versus drug-eluting stent in STEMI. *Rev. Esp. Cardiol. Engl. Ed.* **2014**, *67*, 531–537. https://doi.org/10.1016/j.rec.2014.01.024.
- Bosiers, M.; Deloose, K.; Callaert, J.; Verbist, J.; Hendriks, J.; Lauwers, P.; Schroë, H.; Lansink, W.; Scheinert, D.; Schmidt, A.; et al. Superiority of stent-grafts for in-stent restenosis in the superficial femoral artery: Twelve-month results from a multicenter randomized trial. *J. Endovasc. Ther.* **2015**, *22*, 1–10. https://doi.org/10.1177/1526602814564385.
- Chamié, D.; Almeida, B.O.; Grandi, F.; Filho, E.M.; Costa, J.R.; Costa, R.; Staico, R.; Siqueira, D.; Feres, F.; Tanajura, L.F.; et al. Vascular response after implantation of biolimus A9-eluting stent with bioabsorbable polymer and everolimus-eluting stents with durable polymer. Results of the optical coherence tomography analysis of the BIOACTIVE randomized trial. *Rev. Bras. Cardiol. Invasia* **2015**, *23*, 28–37. https://doi.org/10.1016/j.rbci.2015.02.001.
- Sia, J.; Nammas, W.; Niemelä, M.; Airaksinen, J.K.E.; Lalmand, J.; Laine, M.; Tedjokusumo, P.; Nyman, K.; Biancari, F.; Karjalainen, P.P. Gender-based analysis of randomized comparison of bioactive versus everolimus-eluting stents in acute coronary syndrome. *J. Cardiovasc. Med.* **2015**, *16*, 197–203. https://doi.org/10.2459/JCM.0000000000000086.
- Karjalainen, P.P.; Airaksinen, J.K.E.; de Belder, A.; Romppanen, H.; Kervinen, K.; Sia, J.; Laine, M.; Nammas, W. Long-term outcome of early percutaneous coronary intervention in diabetic patients with acute coronary syndrome: Insights from the BASE ACS trial. *Ann. Med.* **2016**, *48*, 376–383. https://doi.org/10.1080/07853890.2016.1186829.
- Karjalainen, P.P.; Niemelä, M.; Pietilä, M.; Sia, J.; de Belder, A.; Rivero-Crespo, F.; de Bruyne, B.; Nammas, W. 4-Year outcome of bioactive stents versus everolimus-eluting stents in acute coronary syndrome. *Scand. Cardiovasc. J.* **2016**, *50*, 218–223. https://doi.org/10.1080/14017431.2016.1177198.
- Kayssi, A.; Al-Atassi, T.; Oreopoulos, G.; Roche-Nagle, G.; Tan, K.T.; Rajan, D.K. Drug-eluting balloon angioplasty versus uncoated balloon angioplasty for peripheral arterial disease of the lower limbs. *Cochrane Database Syst. Rev.* **2016**. https://doi.org/10.1002/14651858.CD011319.pub2.
- Varho, V.; Kiviniemi, T.O.; Nammas, W.; Sia, J.; Romppanen, H.; Pietilä, M.; Airaksinen, J.K.; Mikkelsson, J.; Tuomainen, P.; Perälä, A.; et al. Early vascular healing after titanium-nitride-oxide-coated stent versus platinum-chromium everolimus-eluting stent implantation in patients with acute coronary syndrome. *Int. J. Cardiovasc. Imaging* **2016**, *32*, 1031–1039. https://doi.org/10.1007/s10554-016-0871-7.
- Karjalainen, P.; Paana, T.; Ylitalo, A.; Sia, J.; Nammas, W. Optical coherence tomography follow-up 18 months after titanium-nitride-oxide-coated versus everolimus-eluting stent implantation in patients with acute coronary syndrome. *Acta Radiol.* **2017**, *58*, 1077–1084. https://doi.org/10.1177/0284185116683573.
- Karjalainen, P.P.; Nammas, W. Titanium-nitride-oxide-coated coronary stents: Insights from the available evidence. *Ann. Med.* **2017**, *49*, 299–309. https://doi.org/10.1080/07853890.2016.1244353.
- Karjalainen, P.P.; Niemelä, M.; Laine, M.; Airaksinen, J.K.E.; Ylitalo, A.; Nammas, W. Usefulness of Post-coronary Dilation to Prevent Recurrent Myocardial Infarction in Patients Treated with Percutaneous Coronary Intervention for Acute Coronary Syndrome (from the BASE ACS Trial). *Am. J. Cardiol.* **2017**, *119*, 345–350. https://doi.org/10.1016/j.amjcard.2016.09.057.
- Nammas, W.; Airaksinen, J.K.E.; Romppanen, H.; Sia, J.; de Belder, A.; Karjalainen, P.P. Impact of Preexisting Vascular Disease on the Outcome of Patients with Acute Coronary Syndrome: Insights from the Comparison of Bioactive Stent to the Everolimus-Eluting Stent in Acute Coronary Syndrome Trial. *Angiology* **2017**, *68*, 513–518. https://doi.org/10.1177/0003319716664266.
- Nammas, W.; de Belder, A.; Niemelä, M.; Sia, J.; Romppanen, H.; Laine, M.; Karjalainen, P.P. Long-term clinical outcome of elderly patients with acute coronary syndrome treated with early percutaneous coronary intervention: Insights from the BASE ACS randomized controlled trial: Bioactive versus everolimus-eluting stents in elderly patients. *Eur. J. Intern. Med.* **2017**, *37*, 43–48. https://doi.org/10.1016/j.ejim.2016.07.027.
- Varho, V.; Nammas, W.; Kiviniemi, T.O.; Sia, J.; Romppanen, H.; Pietilä, M.; Airaksinen, J.K.; Karjalainen, P.P. Comparison of two different sampling intervals for optical coherence tomography evaluation of neointimal healing response after coronary stent implantation. *Int. J. Cardiol.* **2017**, *227*, 194–200. https://doi.org/10.1016/j.ijcard.2016.11.173.
- Hsu, C.C.T.; Kwan, G.N.C.; Singh, D.; Rophael, J.A.; Anthony, C.; van Driel, M.L. Angioplasty versus stenting for infrapopliteal arterial lesions in chronic limb-threatening ischaemia. *Cochrane Database Syst. Rev.* **2018**. https://doi.org/10.1002/14651858.CD009195.pub2.
- Hernandez, J.M.D.; Moreno, R.; Gonzalo, N.; Rivera, R.; Linares, J.A.; Fernandez, G.V.; Menchero, A.G.; del Blanco, B.G.; Hernandez, F.; Gonzalez, T.B.; et al. The Pt-Cr everolimus-eluting stent with bioabsorbable polymer in the treatment of patients with acute coronary syndromes. Results from the SYNERGY ACS registry. *Revasc. Med.* **2019**, *20*, 705–710.
- Kayssi, A.; Al-Jundi, W.; Papia, G.; Kucey, D.S.; Forbes, T.; Rajan, D.K.; Neville, R.; Dueck, A.D. Drug-eluting balloon angioplasty versus uncoated balloon angioplasty for the treatment of in-stent restenosis of the femoropopliteal arteries. *Cochrane Database Syst. Rev.* **2019**. https://doi.org/10.1002/14651858.CD012510.pub2.
- Kuroda, K.; Otake, H.; Shinohara, M.; Kuroda, M.; Tsuda, S.; Toba, T.; Nagano, Y.; Toh, R.; Ishida, T.; Shinke, T.; et al. Effect of rosuvastatin and eicosapentaenoic acid on neoatherosclerosis: The LINK-IT Trial. *EuroIntervention* **2019**, *15*, E1099–E1106. https://doi.org/10.4244/EIJ-D-18-01073.
- Collet, C.; Tonino, P.A.L.; Mizukami, T.; Pijls, N.H.J.; De Bruyne, B.; Karjalainen, P.P. Reply: The Randomized TIDES-ACS Trial. *JACC Cardiovasc. Interv.* **2020**, *13*, 2444–2445. https://doi.org/10.1016/j.jcin.2020.09.009.
- Kuno, T.; Takahashi, M.; Hamaya, R. The Randomized TIDES-ACS Trial. *JACC Cardiovasc. Interv.* **2020**, *13*, 2444. https://doi.org/10.1016/j.jcin.2020.08.004.
- Wardle, B.G.; Ambler, G.K.; Radwan, R.W.; Hinchliffe, R.J.; Twine, C.P. Atherectomy for peripheral arterial disease. *Cochrane Database Syst. Rev.* **2020**. https://doi.org/10.1002/14651858.CD006680.pub3.
- Gómez-Lara, J.; Oyarzabal, L.; Brugaletta, S.; Salvatella, N.; Romaguera, R.; Roura, G.; Fuentes, L.; Pérez Fuentes, P.; Ortega-Paz, L.; Ferreiro, J.L.; et al. Coronary endothelial and microvascular function distal to polymer-free and endothelial cell-capturing drug-eluting stents. The randomized FUNCOMBO trial. *Rev. Esp. Cardiol. Engl. Ed.* **2021**, *74*, 1013–1022. https://doi.org/10.1016/j.rec.2021.01.007.
- Gomez-Lara, J.; Oyarzabal, L.; Ortega-Paz, L.; Brugaletta, S.; Romaguera, R.; Salvatella, N.; Roura, G.; Rivero, F.; Fuentes, L.; Alfonso, F.; et al. Coronary endothelium-dependent vasomotor function after drug-eluting stent and bioresorbable scaffold implantation. *J. Am. Heart Assoc.* **2021**, *10*, e022123. https://doi.org/10.1161/JAHA.121.022123.
- Sia, J.; Nammas, W.; Collet, C.; De Bruyne, B.; Karjalainen, P.P. Comparative study of neointimal coverage between titanium-nitric oxide-coated and everolimus-eluting stents in acute coronary syndromes. *Rev. Esp. Cardiol.* **2023**, *76*, 150–156. https://doi.org/10.1016/j.recesp.2022.05.011.
